# Culture and Green Advertising Preference: A Comparative and Critical Discursive Analysis

**DOI:** 10.3389/fpsyg.2020.01944

**Published:** 2020-09-18

**Authors:** Shubo Liu, Xiaoyuan Liu

**Affiliations:** Business School, Central University of Finance and Economics, Beijing, China

**Keywords:** green advertisement, critical discourse analysis, corporate environmentalism, socio-cognition, environmental protection

## Abstract

As environmental concerns began to emerge, companies started to target toward the growing ‘green market’ to launch their green products. Companies’ green advertising played an important role in facilitating corporate green marketing and fuelling the desire for environmental-friendly commodities. Applying a Critical Discursive Perspective, this study focuses on corporate environmental advertising in order to illuminate their discursive strategies and the process through which corporate green advertising generates and symbolically structures the necessity of green consumption. The comparison of constructive characteristics and constructed meanings of green advertisings identified embody distinction in Western and Chinese cognitive style and process.

## Introduction

Public concerns over environmental issues have produced a dramatic increase in the introduction of ‘green’ or environmentally friendly products, and many companies are engaged in environmental marketing (Bahn and Wright, 2001; Leonidou et al., 2011; Dangelico and Vocalelli, 2017). In this context, corporate green advertising has emerged to manifest the combination of the globalized ‘green movement’ and corporate marketing. ‘Green advertising’ is defined as commercial advertising that uses an environmental theme to promote products, services, or corporate public images (Banerjee et al., 1995). In developing economies such as China, marketers are also beginning to make an effort to target the increasingly lucrative green segment of the Chinese population (Chan, 2004). Like their counterparts in the West, these ‘green pioneer firms’ rely on environmental advertising to communicate the eco-friendly aspects of their green products.

Green advertising, by claiming its promoted brand or product as ‘green,’ seems to have removed its negative environmental impacts. But a growing number of studies (e.g., Carvalho, 2001; Banerjee, 2003; Alcott, 2005; Bäckstrand and Lövbrand, 2006; Böhm and Brei, 2008; Raineri and Paillé, 2016) have suggested that it is still not a fact that business has become reconciled with the environment. Therefore, commercial green advertising is likely to reconcile the conflicting demands of environmental protection and business production and consumption. The corporate green advertising discourse connects consumerism to environmentalism and seems to deliver consumers a particular type of commercialized environmentalism.

To be more specific toward this research context of China, it has been observed that green advertising is rising phenomenally, despite it being at its beginning stage in this emerging economy (Zhu and Sarkis, 2016). However, Chinese cultural context differs from the Western one (Hall, 1955, 1992; Hofstede, 2001), and existing studies have suggested that the environmental protection institutions as well as Chinese people’s understanding and cognition of the “environment” vary from its Western counterpart (Nisbett et al., 2001; Tsai, 2001; Weller, 2006; Child et al., 2007). For example, as Corbett (2006) contends, the social construction of nature or the definitions and meanings, which people tend to build through social interactions about nature, can be quite different from culture to culture. Similarly, and more specifically, as psychologist Chiu (1972) puts it: ‘Chinese are situation-centered. They are obliged to be sensitive to their environment. Americans are individual-centered. They expect their environment to be sensitive to them. Thus, Chinese tend to assume a passive attitude while Americans tend to possess an active and conquering attitude in dealing with their environment (p. 236). Therefore, it can be proposed that in green advertising, firms tend to adjust their environmental messages to their target audience according to cultural preferences. And the representation of the ‘greenness’ constructed by firms operating in China is likely to be influenced by Chinese culture and cognitive style. Consequently, as both multinational corporations and Chinese indigenous companies are launching their green products and producing green advertisings in Chinese markets, the discourses of their green advertisings might be featured differently (Morren and Grinstein, 2016). In the context of China where the idea of commercial environmentalism or green consumption is emerging and is transported from the Western cultural background, it is still not clear how green consumption is advocated for, how consumption practices are connected to environmental protection, and how the meaning of green consumption is constructed by firms operating in China.

In such context, the main focus of this study is on discourses active on corporate websites as an advertising channel in the digital era, and aims to explore in what ways companies use both texts and visuals to represent their green products and themselves as environmentally responsible. We aim to answer the research question: what discursive strategies have been applied in constructing the meaning of commercial environmentalism or green consumption? Are there any differences in the advertising discourses from Chinese and Western backgrounds?

## Literature Review

### Research on Green Advertising

The developments in green advertising practices have drawn the attention of marketing scholars who follow different research streams (Leonidou and Leonidou, 2011). They aim to research on various aspects of green advertising, such as its nature, structure, content, effect, and development trends (Leonidou and Leonidou, 2011). One dominant stream of green advertising research focuses on the advertising effects. There are mainly two angles examining green marketing and advertising literature: one focuses on green marketers and the advertisement itself from the company side, as reviewed above; and the other focuses on green consumers or the relationship between advertisers and advertisement receivers. The effect of environmental advertisings on consumer behavior is thus the focus of another stream of green advertisement studies. In this stream of study, mainly in a quantitative approach, researchers focus on consumers’ responses to and attitudes toward green advertisings, and find out which kinds of green advertising appeals are the most effective for consumers (e.g., D’Souza and Taghian, 2005; Chan et al., 2006).

Among the scant studies of green marketing and advertising in China, Chan and Lau (2000) conducted surveys to study the antecedents of green purchases in China’s green market. Chan (2004) examined Chinese consumers’ responses to environmental advertising. Dai et al. (2011) studied the development of green advertising in the Chinese automobile industry. They propose that the government of China should use extensive propaganda imperative policies to enhance the “green consciousness” of the people and the companies, because environmental awareness is the precondition to green consumption. Similarly, Chan and Lau (2000) propose that the green marketers should:

“[…] increase their investment in consumer education so as to further raise the environmental consciousness of their target customers […] they should consider sponsoring environmental education in schools, and forming alliances with the government and/or environmental groups to promote the ethics of “green” consumption through various propaganda vehicles, such as television and radio broadcasts, exhibitions and seminars.” (p. 307)

Moreover, green advertising in different forms has been commonly found in China (Chan, 2004). Ye’s (2000) survey indicates that more than 90% of surveyed firms are interested in selling green products in the Chinese market, and in the market there have already been about 3,000 different kinds of eco-friendly products. Ongkrutraksa’s (2003) study shows that the majority of advertisers in China seek to use mostly green messages (promotion of a green company image) and attempt to project a green corporate image, rather than focus on the environmental benefit of their product or service. The existing literature on Chinese green advertising, however, mainly stays at a surface level and focuses on the constitutive factors in the content of green advertisings, while the constructive characteristics and constructed meanings of green advertising discourse have not been paid attention.

### The Constructive Role of Green Advertising

In Discourse Theories, language/discourse not only exists in a structural form, but also as constructive process. Laclau and Mouffe (1985) modify the structuralism linguistics of Saussure (1960) in line with the post-structuralist view and treat language use as alterable through the day-to-day interactions of social actors. Laclau and Mouffe (1985) argue against the study of language as a fundamentally synchronic entity, since (in terms of the metaphor) signs cannot be fixed definitively into position. Instead, the position of signs is always up for negotiation, and it is this constant negotiation of meaning that accounts for the contingency of discourses. However, despite their rejection of Saussurian principles, Laclau and Mouffe (1985) retain the notion that signs strive to acquire fixed meaning from their relation to one another. They argue that, although this is ultimately impossible, discourses attempt to fix signs into certain positions. Discourse analysis – as understood here – attempts to map out the constructive processes by which the meaning of signs can become relatively fixed (and unfixed).

Aligning with structuralism assumption, there has been quantitative content research exploring the characteristics of green advertising content. For example, researchers applying content analysis explored the functional dimension of green advertisings and examine if the green advertising can induce consumers’ purchase decisions (McGowan, 2000). On the consumer side, such studies aim to measure consumer attitudes toward green advertising and environmental attitudes (e.g., Haytko and Matulich, 2008; Casado-Aranda et al., 2018). In this paradigm of research, the green advertising is assumed to be an instrumental role and the method of content analysis is mostly used to comprehend the nature of green advertisements, namely their composition and functionality. However, the structuralist approach faces difficulties in explanations on the construction characteristics and constitutive components of the green advertisings. Noticing the incompleteness, other scholars seeing advertising in its active form examine the phenomenon for advertising’s meanings by using the constructive role of advertising and its relationship with the subjectivity of consumers. For example, via a qualitative and discursive research approach, Garland et al. (2013) and Chen (2016) studied the configuration features of hybrid car advertisings. Such studies found that using ambiguous messages in green advertising can promote socially and politically charged products for consumers’ understanding and imagination. Similarly, drawing on findings from a rhetorical analysis of advertising and branding efforts by an environmentally conscious cleaning product company, Ryan (2012) claimed that the role of advertising was shifting: the advertisements nowadays, besides disseminating material lifestyle aspirations and product information, have been utilized as ‘Agenda-Setting socio-political tools’ enabling private firms to incorporate social issues, such as the environmental movement, into their advertising messages (Ryan, 2012, p. 73).

The above perspective assumed that companies hold an active stance to shape their legitimacy through communication and, thereby, influence public perceptions. In this respect then, corporate green advertising as a way of corporate communication, can be seen as ‘a public relations vehicle’ aimed at influencing people’s perceptions (e.g., Dutton and Dukerich, 1991; Hooghiemstra, 2000). This stream of research on corporate advertising assumes an active and constructing role. However, corporate advertising’s cultural/political role and its semiotic effect have been neglected in the field of green marketing and advertising research. As Caruana and Crane (2008) and Glozer et al. (2014) pointed out, to date, the majority of studies on consumer responsibility have relied on the assumption that responsibility is ‘an objectively identifiable trait of sovereign consumers’ (Caruana and Crane, 2008), despite the recently emerged research focusing on the discursive and cultural aspect of corporate advertising and communication (e.g., Böhm and Brei, 2008; Saint, 2008; Hansen, 2010; Eyles and Fried, 2012; Ryan, 2012; Tregidga et al., 2014). This research will adopt a critical discursive perspective to study green advertising and consider the interaction between discursive strategies and their cultural contexts.

### The Discourse of Green Advertising in Contexts

In the critical discursive perspective, green advertising as an active discourse represents an attempt to fix a web of specific meanings within a particular domain, and it is developed in different social contexts and in a specific manner which will keep the needs of certain social actors. To be more specific, the discourse of green advertising articulates people’s cognition and understanding of the natural environment. And the discourse should be treated as heterogeneous and its embodied meanings as diversified. As Banerjee et al. (1995) claim, although the green discourse was initially rooted in environmental activism, it has undergone semantic broadening and disseminated through other domains of public discourse (such as social media and corporate marketing). In this sense and in lots of cases, the meaning of ‘greenness’ has been extended, or even transformed, and thus appears much less evident in its link to environmental issues.

Following Mühlhäusler and Peace (2006), ‘green discourse’ is defined as environmental discourse comprising linguistic devices articulating arguments about the relationship between humans and the natural environment. There is also a variety of environmental management and governance discourse as green discourse. From a political viewpoint, Bäckstrand and Lövbrand (2006) argue that environmental discourse can be generally categorized as ecological modernization, green governmentality, and civic environmentalism. Each category of green discourse has different perspectives toward environmental problem solving and environmental protection. These green discourses have an impact on corporate green advertising.

It is expected that corporate green advertising discourse recruits elements from existing meta-green discourses and cultural contexts, but it is not clear whether these discourses vary and correlate with cultural differences and in what way such discursive elements are arranged into corporate green advertising, and how companies make efforts to contribute to the commercial meaning of ‘green’ through their green advertising. Especially in the Chinese social context, both the industrial development and people’s cognition of the natural environment are very different from their Western counterparts (Nisbett et al., 2001; Tsai, 2001; Weller, 2006). Regarding the perception of relationship between human beings, the natural environment, and environmental protection issues, Westerners are expected to perceive more control in a given situation (solving environmental problems) than do East Asians, and a greater expectation of success when the self is involved in interaction with the environmental problems (Langer, 1975). In addition, insomuch as harmony remains the watchword in social life and relations for Chinese people, a compromise and holistic solution to environmental problems is more sought after (Nisbett et al., 2001). More generally, cultural differences are observed in terms of cultural dimensions (Hofstede, 2001). Similarly, Hall (1992) has pointed, in a high-context culture, there are many contextual elements that help people to understand the rules, while in a low-context culture, very little is taken for granted. Such distinctions in cultural factors and socio-cognitive differences might pose an influence to the discursive production and strategies of green advertising.

Moreover, it can be hypothesized that green advertising discourse as a sub-category of commercial discourse bears the same characteristic of practicality. However, as mainstream advertising is aimed at promoting consumption and thus potentially involved in materialism, and materialism is intrinsically contradictory to environmentalism (Banerjee and McKeage, 1994), how do firms compromise the conflict in their green marketing discourse? In addition, as discourse plays a vital role in representing firms’ green brands and products, and constructing meanings of green consumption for consumers, and commercial advertisings can be seen as a manifesto of companies’ perceptions toward environmental issues, it is important to understand the representation of commercial greenness. In sum, the culturally embedded discursive process of firms constructing the meanings of greenness through their advertisings, as well as this process of how green advertising helps firms play their authoritative role in the construction of eco-knowledge and informing consumption practices dealing with environmental degradation is worth investigating.

## Methodology – Critical Discourse Analysis

In line with Fairclough’s (1992) argument that discourses are constrained by and situated in social contexts, as well as both reflecting contexts and constituting them, this study tries to comprehend how language is used in a given context, such as social structures, cultural norms, and physical legacies that discourse occurs within. Based on such arguments, our research adopts the critical discourse analysis (CDA) approach. CDA approach aims to systematically explore often opaque relationships of causality and determination between (a) discursive practices, events, and texts, and (b) wider social and cultural structures, relations and processes; to investigate how such practices, events and texts arise out of and are ideologically shaped by relations of power and struggles over power; and to explore how the opacity of these relationships between discourse and society is itself a factor securing power and hegemony” (Fairclough, 1993, p. 135).

**FIGURE 1:**
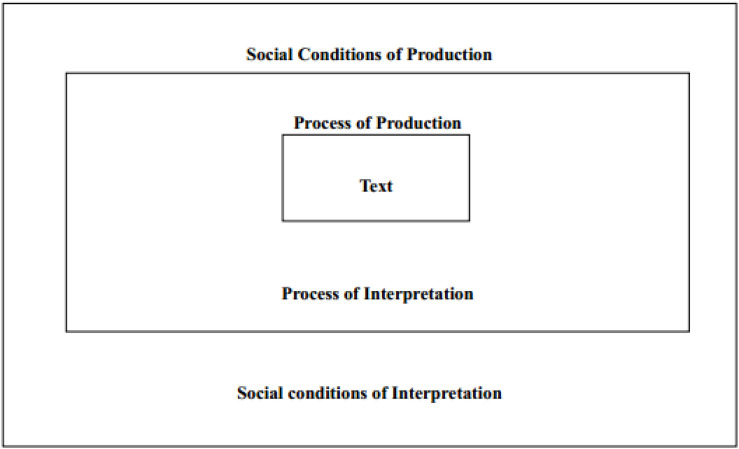
Fairclough’s three-dimensional model (Fairclough, 2001, p. 21). Adopted from: Fairclough (2001).

Based on a CDA perspective, this study provides a systematic set of inquiries to analyze both textual and visual constructs in relation to social phenomena. It makes an effort to understand how companies use language, and explores the types of messages that firms communicate via websites. Methodologically, Fairclough (1992;) provides a three-dimensional analytic framework which is sensitive to the social/cultural contexts of discourse (see Figure 1).

Fairclough’s CDA approach focuses on studying discursive events; an event is an “instance of language use, analyzed as text, discursive practice, and social practice” (Fairclough, 1993, p.138). Therefore, a discursive event involves both texts, discursive practices (production and interpretation of the text), and social practices (including situational, institutional and societal practice).

Based on this idea and within a critical discourse tradition, Fairclough in his several influential works including Language and Power (1989), Discourse and Social Change (1992), and Critical Discourse Analysis (1995), proposed a three-dimensional framework that could be employed to relate micro levels of language use to wider aspects of social practice. Social practice can be analyzed using the construct of “order of discourse,” which refers to the sum of all discourses that are in practice within a specific social domain or institution (such as the media, or university, or in this study, corporate advertising; Fairclough, 1993). Every communicative or discursive event consists of three dimensions – text, discursive practice and social practice – and should be analyzed accordingly:

(1) Text: the linguistic features of the text, including lexicalisation, grammar, cohesion, and text structure.
(2) Discursive practice: processes related to the production and consumption of the text, including the “force” of utterances, coherence, intertextuality, and interdiscursivity.
(3) Social practice: the institutional and organizational circumstances of the discursive event and the constitutive effects of discourse.

### Data Collection

The World Wide Web offers a great scope for companies to manage their public relations and sales promotion. As Chaffey et al. (2000, p. 42) put it: “So a company having its own web site becomes its own media owner and has the opportunity to publish any kind of material without an intermediate review from a publisher or television company.” Such self-presenting features suit these research intentions. This research is not intended to study if the corporate website is the most suitable marketing medium, but to focus on the construction of corporate greenness in advertising discourses. Thus, corporate website discourse ought to be the best data source since it is purely from the firms without any filtering processes involved by other parties such as publishers or television companies.

A purposeful sampling technique is adopted in this study. Purposeful sampling allows the researcher to select samples with intention. Such selection method allows the researcher to focus in detail on a certain issue, subject, or phenomenon (Patton, 2002). According to Patton (2002, p. 230): “the logic and power of purposeful sampling lie in selecting information-rich cases for study in depth.” Previous studies (Creswell, 2003) have proved that the idea of purposeful sampling is appropriate for investigations on online materials.

In order to select the most suitable samples, companies are selected according to the following four criteria: firstly, the company should have a series of green products (products are communicated as having environmental protection features, such as pollution reduction or energy efficiency enhancement), and should have launched its green campaigns for advertising their green products in the Chinese market. Secondly, the firms should be from resources-based industries which have received the most environmental pressure and have had prominent environmental impact. Such industries can be real estate development, automobile manufacturing, chemical industry, or machinery manufacturing. Thirdly, the company should have a strong environmental performance in its industry and should have been rated as the top green firms in China for consecutive years (from 2014 to 2018) according to China’s Green Company rating. Lastly, the green products/services of the firm should be profitable and thus successful in the market.

Based on the selection criteria, four companies that advertise their environmentally friendly product through corporate websites have been selected for data collection: general electric (GE) in China, Unilever in China, BiYaDi (BYD) Auto, and Landsea Real Estate. These four companies are categorized into two groups for comparative analysis: Category One as multinationals (MNCs) subsidiaries (GE and Unilever) and Category Two as Chinese indigenous companies (BYD and Landsea). To study these corporate websites in detail, we focus on discourse from Products/Services Introduction page, as well as the “green” content from their Home, Introduction, and Sustainability/corporate social responsibility (CSR) web pages. In addition to textual information, visual information from product introduction pages and from webpage embedded video clips was collected and analyzed. In sum, there are 76 advertising samples collected for analysis (Table 1).

**TABLE 1:** Advertisements collected from websites.

**Firms**	**Forms of Advertisings**	**Sources/Number of the Advertisings**				**Total Number: 76**
		**Home**	**About/Intro**	**CSR/Sustainability**	**Products**	
GE	Textual	1	3	1	6	11
	Visual	0	2	1	4	7
	Multimodal	1	0	0	3	4
Unilever	Textual	1	1	2	2	5
	Visual	0	0	0	4	4
	Multimodal	2	0	1	1	4
BYD	Textual	1	1	0	0	2
	Visual	0	2	0	0	2
	Multimodal	1	1	4	8	14
Landsea	Textual	0	0	1	2	3
	Visual	0	0	0	5	5
	Multimodal	4	3	0	7	14

The unit of advertisement samples should be either an advertisement introducing a green product or technology in several textual paragraphs, or an excerpt from a Home Page news report focusing on the company’s new green achievement, or an advertisement presented by multimodal discourses including both textual, visual, and vocal information (such as a video clip embedded in the web site), or one paragraph introducing the company’s environmental responsibility and its overall green businesses, or simply one screenshot picture presenting an array of corporate products, activities of corporate actors, and links to additional content, single pictures (especially from Home Pages) or an image with inserted texts.

The data set is large enough to allow the identification of patterns but small enough to reveal multiple, rich levels of meaning, as in, for example, Fairclough’s study on the privatization of public universities. In addition, the two categories of data source allow us to have a comparative approach which adds a valuable perspective to our understanding of the sample advertisements and help to develop conceptual themes in the green advertisements.

## Data Analyses and Findings

### The Descriptive/Textual Analysis

The Product Introduction Page provides detailed textual information on firms’ green products and services. The promotional texts of the product advertisings not only stress the ‘environmental-friendly’ facet, but also emphasize the facet of ‘hi-technology’ in their green products. Such technological advancement is always linked to innovation, improved efficiency and economic advantages. Additional functional facets of green products are also often found in the discourse, such as ‘safety,’ ‘convenience,’ and ‘coziness.’

In addition, new green words/terms have been coined by the MNCs to name their green products/service or green projects. For example, GE coins the word of ‘*ecomagination*’ and Unilever brings forward its ‘Sustainability Living Plan.’ In the Product page of GE’s website, all green products are introduced as a subfield category under the main theme of ‘*ecomagination*.’ GE launched their ‘*ecomagination*’ campaign in 2005 in order to promote their energy-efficient technology products and services, and to construct the company’s public image as a leading socially responsible company. In comparison, the Chinese firms are less active in ‘green vocabulary.’ In their advertised product names, the green factors are literally presented. For example, the advertised green cars from BYD are named as ‘*pure electric car e6*,’ ‘*DM dual-mode electric car*,’ and ‘*K9 pure electric bus*’.^1^; accessed chousing products according to their different market sectors, such as ‘*green house for first-time house buyers*’ and ‘*green residence for the aged people*’.^2^

### The Descriptive/Visual Analysis

In addition to textual information, visual information can be identified. A typical example is from GE’s ‘Wind Power’ (Exhibit 1). The picture of the green product features a close-up shot of a beautiful view of nature. In the foreground, clean and trimmed grasses, standing in the grassland, wave gently in the breeze. In the background, the sky is blue and clear. The two wind turbines stand on the horizon and the line between the grassland and the sky. All the features combined together in this advertising signify a harmonious relationship between the life on Earth and ‘wind power’ generated by human technology. Besides, as the human made participants (two wind turbines) are placed behind the other visual participants (natural objects: trees, grasses), this sequencing of information suggests a sequencing of importance (Kress and van Leeuwen, 2006).

**Exhibit 1:**
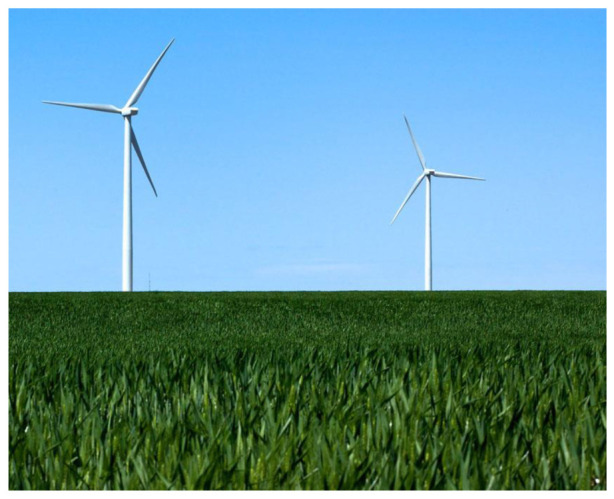
‘Wind Turbines’ Ad 1 for GE. Websites addresses: https://www.ge.com/news/reports/ecomagination-ten-years-later-proving-efficiency-economics-go-hand-hand^3^; http://www.ge-energy.com/wind^4^. Image is from companies’ public advertisement online and the permission of publication of advertising images has been obtained.

A simple semiotic reading would argue that this ad tries to construct a utilitarian fantasy of technology putting natural resources to use. These two wind turbines represent the scientific power which intrudes into the natural territory and frames it as a resource for human use. The sky and the invisible wind which are made visible through the presentation of gently waving grasses are presented as a tamed object of consumption, capable of providing ‘*proven performance, availability and reliability.*’ It also turns into a commercial and privatized sense which brings ‘*more value for our customers.*’ Corbett (2006) illustrates this point of green advertisement: ‘Advertising commodifies the natural world and attaches material value to non-material goods, treating natural resources as private and ownable, not public and intrinsic’ (p. 146).

However, the green products and technology here in the advertising are structured a little differently from the usual fashion, especially compared with the portrayal of technology which appears in traditional advertising or advertising from Chinese local companies (which will be presented later). In this green advertisement, the intrusion of human technology, the two wind turbines, is played down and naturalized by placing them into the secondary position to the primary natural landscape in the foreground and background (see Exhibit 1). This is very different from many other new technology product advertisements in which technology or the product, as well as their function description, is usually represented in the central position of the advertising.

The setting in the GE green advertising also tries to de-materialize the technology and green product – the wind turbine – by presenting simplicity in the visual composition: there is neither sophisticated technological description nor information about the products. Instead, the wind turbines in the picture look like natural objective. Similarly, another advertising picture of the wind turbine is positioned together with coconut trees (see Exhibit 2); the contrast between GE’s products and the natural trees sends a message to the audience: the green product is just another object in the eco-system, same as the trees standing on the sea, and it causes no harm to the natural environment. This parallel strategy, together with the overall campaign theme of ‘ecomagination,’ can be read as a corporate defense against the environmentalist critique of technology – how could the green technology such as the wind turbine possibly cause harm to nature if it can exist in such a perfect and tranquil scene?

**Exhibit 2:**
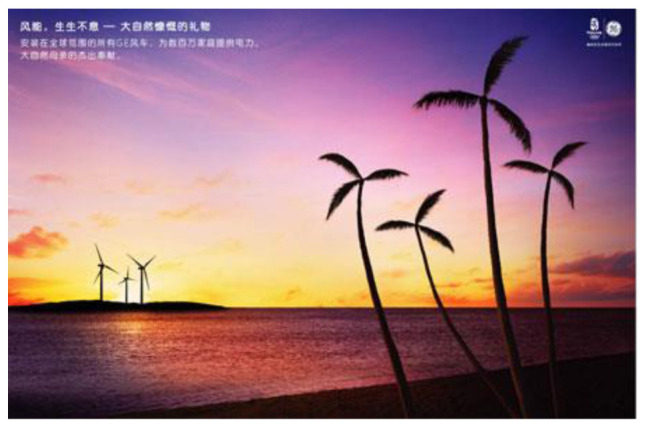
‘Wind Turbines’ Ad 2 for GE. Website address: http://www.ge-energy.com/about/index.jsp^5^. Image is from companies’ public advertisement online and the permission of publication of advertising images has been obtained.

A very similar presentation of the green product from Unilever can be found in Unilever’s green product (see Exhibit 3). The product is even invisible in this advertising; the purifying effect of the product is visually shown in a purifying process, and the ‘U’ shape water represents the brand of Unilever. The invisibility of advertised products further reduces people’s concern about the technological intrusion into the natural environment – how can technology cause harm if it has nothing but a purifying effect?

**Exhibit 3:**
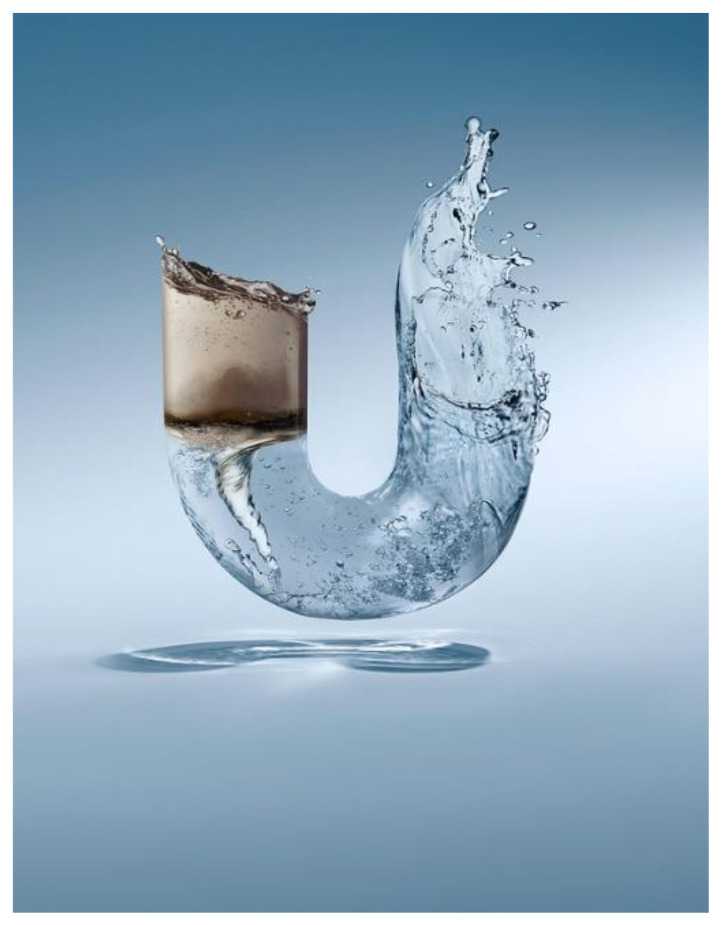
Unilever’s Water Purifier Ad 1. Website address: https://www.unilever.com.cn/sustainable-living/^6^. Image is from companies’ public advertisement online and the permission of publication of advertising images has been obtained.

In addition to the ‘de-materialization’ strategy of representation, a promotional nature of advertising discourse is reflected in its effect of decoration which glorifies the promoted corporate greenness. For example, GE’s ‘ecomagination’ campaign advertising videos represent the utopian version of corporate green advertising which are replete with of ‘imagination,’ ‘invention,’ ‘ideas,’ etc.

Another example is from the visual presentation. Exhibit 4 shows two advertisings of BYD’s pure electric bus, the K9, and the pure electric car, the e6. In these advertisements, the car’s physical presence takes up nearly 1/3 of the advertisement (the bus takes 1/2) and is in the very central position. Its chrome outlook appears shiny and sleek. It is also surrounded by radiating ‘swoosh’ lines, suggesting extreme speed. The fluorescent lines with the light green of the enveloping city background make the green bus/car look technologically advanced; the green leaves decorating and surrounding the bus/car replace its emission pollution.

**Exhibit 4:**
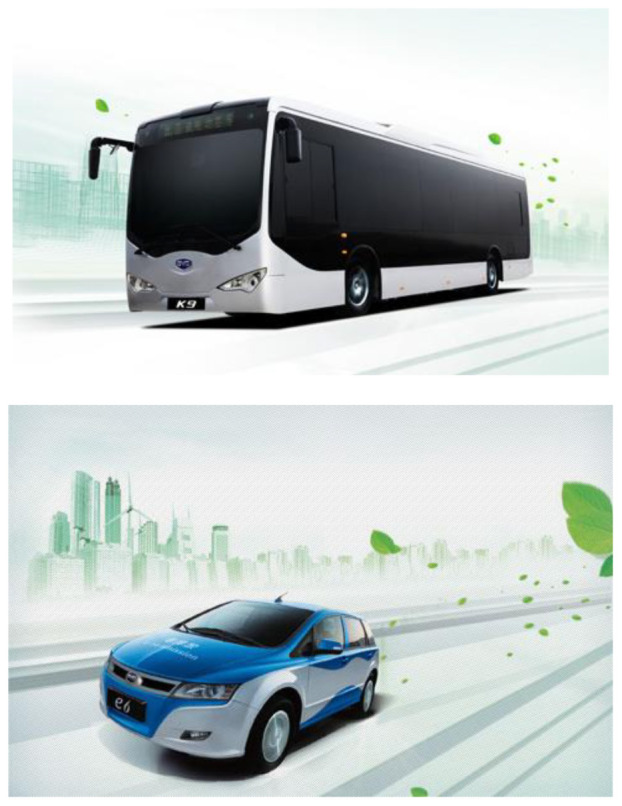
BYD’s Pure Electric Bus ‘K9’ and ‘e6.’ Website address: http://www.bydauto.com.cn/energy.html. Image is from companies’ public advertisement online and the permission of publication of advertising images has been obtained.

Coupled with the streamline design of the bus, these features suggest futurism – a significant Western artistic style employed in, for example, painting, film, architecture, industrial design, and fashion. Futurism stresses speed, technology, youth, and the triumph of humanity over nature (Marinetti, 1973).

Thus, one can see that the environmental value of the electric is rarely represented in the product introduction texts. In the abstract sketch of the background of the advertising, one sees a slightly green city outline and bright sky; the environmental protection factor is positioned as a sort of decoration – nature is down-played and only presents itself through the scattered leaves flying around the bus/car driving in the highway.

So, in the sequencing of information which appears in such advertisements, the product itself is presented as ‘high’ while the environmental factors are ‘low’ (Kress and van Leeuwen, 2006). In the background, the fictitious sketch of the city as the car’s embedding environment also help to play down the environment in the background. The same tendency to trivialize environmental values can also be observed in the product introduction: The BYD is called ‘*the car of tomorrow*,’ but the ad does not specify what kind of ‘tomorrow’ it is. Is it the ‘tomorrow’ of environmental harmony? Or economic prosperity? Or technological development? The answer is unclear. But whatever it is, the advertising suggests that it *‘has not stopped amazing the automobile world*.’

These characteristics of the protruding product as central while marginalizing the natural environment can also be found in the green real estate developer’s product advertisements (see Exhibit 5). In the visual part of the advertising, it shows a view overlooking the whole architectural complex. The advertising does highlight the ‘environmental value’ of the housing complex, but the ‘environmental value’ is not aligned to notions of environmental protection or pollution reduction. Instead it shows it as an environmental aesthetic value. The houses are situated at the foot of a green hill and beside a tranquil stream. Ironically, the vast mountain covered by forest and the fantastic view of a clear river in the advertising seems to be unrealistic for ordinary Chinese consumers living an urban life, although the housing product in the ad is targeting them.

**Exhibit 5:**
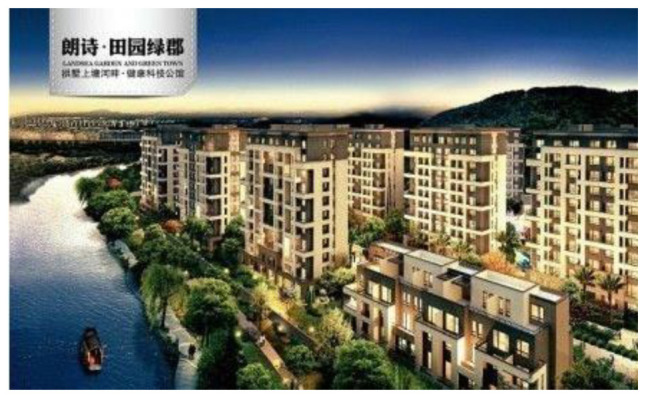
Landsea’s Green Houses: Landsea Countryside-shire. Website address: http://landsea.cn/Group/RealEstate.aspx^7^. Image is from companies’ public advertisement online and the permission of publication of advertising images has been obtained.

In addition, the name of the green building complex (‘*Landsea Countryside-shire*’) is also implying an unrealistic sense of a pleasant bucolic lifestyle. The slogan on the top left of the picture – ‘*healthy technology houses*’ – suggests the functional aspects of the product: it can bring health and housing technologies. Again, the issue of environmental protection is not mentioned.

By calling on the urban or new urban rich to live in the not-yet-polluted land of the rural region or the rural poor, this advertising exacerbates the already serious environmental inequality along class and geographical lines. It seems the green house is presented in the advertising as a way to escape the pollutions of city life: as long as you can afford to buy this ‘green’ house, you will live in a clean environment.

This advertising addresses the public’s increasing environmental concerns, it proposes an extremely individualistic solution – to run away. Escape into pristine nature all by yourself, simply through purchasing an advertised ‘green house.’ In this sense, instead of marketing the house as a solution for saving the environment, the green product is portrayed as a ‘parachute’ or ‘escape pod’ in which the urban rich can flee from pollution. They can then explore their private eco-utopia, which is to be enjoyed alone or/and with families. The utopia portrayed here constructs a fantasy of human nature harmony to cover the rising social anxiety about environmental pollution. And the utopia is envisioned through the expansion of consumption: to consume more and consume the advertised green products.

### Interpretative Analysis – Themes and Discursive Strategies

Referring to Fairclough’s (2001) interpretative analysis framework, discursive aspects such as the contents of the language, the subjects, and the relationship existing among the subjects need to be analyzed in order to decode the tissues of meaning in the narrative construction. Based on descriptive and interpretive analyses of representative green advertisements, three common themes or discursive strategies can be identified (see Table 2).

**TABLE 2:** Common themes and discursive strategies.

**Themes**	**Strategies**
Re-coloring the corporate greenness	***Positioning*** – intertextuality and interdiscursivity
Making sense of the corporate greenness	***Embedding*** – embedding the greenness into the existing discourses; rationalism; futurism
Perfecting the corporate greenness	***Idealizing*** – pacification, topic avoidance, and utopian version

The first discursive strategy is to re-color the greenness. The studied firms are all found to communicate green in a more-than-green way and their green products are not only marketed on their eco-friendliness (to protect the planet earth) but also on attributes such as products’ property of pleasantness, high-technology, fuel efficiency and the likelihood of reduced fuel costs. A few quotes mention the eco-benefits for the environment, and in many cases the green discourse blur the boundary of between conventional and green product; this in order to give more breadth to the idea of commercial greenness, and to fit green products into a hi-tech, and holistic designs which are deemed more about nature than just their ‘greenness.’

The emphasis on traditional features of products over their environmentally friendly features implies that commercial greenness has been embedded in the context of instrumentality.

Along with constructing green products as multi-facets, corporate green advertising discourse defines the green consumer subject, and the texts inform their audience that being green is not just about being responsible for the environment, but entails multiple roles: they are not only consumers, but also ‘environmentally concerned contributors,’ ‘responsible participants,’ and ‘caring family members.’ One subject that companies particularly frame and align with consumers is the value-advocate/contributor/participant/patron of responsible and savvy practices. The semantic power in advertising discourse leads interpretations that the audiences are not just consumers. Below provides a manifest reference in Unilever and Landsea’s green advertisings:

‘In our Sustainability Living Plan, our role as a company is no longer solely in marketplace but also to help society to achieve sustainability development…our green products become a channel to transmit our message and consumers by purchasing our green products can join us for more sustainable living styles.’^8^ (Unilever)

‘Our products stand for both green and humanity; the greenness bears our value on environmental responsibility and a harmonious relationship between human beings and the nature.’^9^ (Landsea)

The texts present value that distance commercial sense, meanwhile they act to construct consumer subjects who choose the products as a responsible and caring citizen and promoter of high-tech, highly personalized and highly responsible value-embedded products.

Secondly, by presenting the technological competitiveness and economic advantages with statistical data, the advertising discourse ostensibly makes sense of and portrays the commercial greenness as a must-take option for consumers by forecasting future standards. In shaping green products as a necessary solution to consumer’s problems and a contributor to everyday life, companies are constructed as the provider of the achievement, thus they obtain power to control what is needed/desired for green consumption and green consumers. Exemplary advertisings are found as:

‘The economic growth is slowing down, which will bring to an end to the golden-10-year growth. And the previous inefficient model of economic growth will be replaced by intensive growth and sustainability development. In the same time, obsolete energy-consuming production and products will give place to green production and green products.’ (BYD)

‘We are not afraid to say it because what we do very much align with China’s next 5 years plan. For example, both energy and healthcare are very important to China; we are hand in hand with China for the next 5 years. What we do, what we are facilitating to help, to make the world better, we can say that because we have proven that we have done so in the past 120 years.’ (GE)

In the above statements, corporate communication discourse strategically poses the environmental and resource-related challenges as undoubted upcoming realities and thus presents their green products as the inevitable choice for the audience. Firms’ green products play a role as savior helping solve clients’ environmental pressure; by relating to and stressing the macro environment threats such as the slow-down of economic growth, the endangering natural environment, and the depletion of resources, being green and consuming greenness seems to be the only choice left. The discursive effect of future tense such as ‘*will*,’ ‘*be going to*’ used in the sentences is to center the green products and green consumption as an essential approach for consumers to achieve this end: choosing greenness means choosing the future. In addition to using the future tense statements which shape greenness as necessary for the future, firms also strategically place discourse which highlights that the greenness has become a fad in society and draw on the contextual background. So appropriating green consumption is necessary.

The promotional consumption discourse produces the possibility of an ‘ideal self’ of consumer culture (Holt and Thompson, 2004; Thompson, 2004) and implies to the audience that being a consumer can also mean being a contributor in environmental protection, as long as consumers choose the advertised green products. And, the more you consume, the more you can contribute to environmental benefits. In this way a subject role of ‘green consumer’ is constructed and the firm is able to connect green products to its brand. The discursive strategy in the above excerpt also helps to solve the tension between consumption and conservation, and the promoted green product is mythologized to turn consumers into contributors to, and protectors of, the environment.

However, in a critical perspective, this constructed subject position concerning greenness is not based on a desire to deal with ecological destruction or to protect the environment itself, but to be ready to live in the house – the green product – to escape from the threat of pollutions. The health-threatening ‘environment’ in the corporate green advertising discourse apparently has been turned into an urge for green consumption, and the green product has been turned into a green protector, shielding consumers from pollution. In this way, green consumption has incorporated into its message, certain other meanings such as guarding family member’s health, and gaining a healthy lifestyle. Therefore, other identities besides the identities of ‘responsible consumers who care about the environment’ are created, and the advertising discourse helps to enlarge the meaning of greenness and frame subjective categories: the subject of caring husband to wife/son to parents/parents to children.

In the end and based on the previous two strategies, a pacification and perfecting strategy emerges. This can be found in the green advertisings shaping a utopian version of the consumption world in which advertised corporate greenness helps to meet environmental challenges, and corporate green product/technology serves as a panacea for environmental threats.

In a nutshell, a common connection exists between corporate green advertising discourse and a broader societal context. The discursive strategies such as positioning, embedding and idealizing (see Table 2) are in order to represent green consumption as a feasible solution to an environmental problem, and a direction to the future, or a green consumption lifestyle. Themes 1 and 2 are about positioning environmental responsibility with green returns (such as functional aspects of green products), and embedding ‘greenness’ into a broader context and making worldly meanings of the corporate greenness (such as economic benefits, people’s health, and technocratic). In such positioning and embedding process through intertextuality and interdiscursivity, people’s direct concern for the environment is diluted and people’s attention redirected to consumptions through the all-around green products. Theme 3 is for idealizing the corporate greenness by constructing utopian versions of a green future. The corporate greenness discourse in a promotional style naturalizes the intrusion of humans’ industrial practices to nature and therefore seems to guarantee a problem-free version of green consumption.

This differs from the traditional environmentalist understanding of ‘greenness,’ which holds the opinion that radical changes to the current lifestyle and economic systems should take place in order to reverse environmental degradation and reduce industrial damages to the nature (Dunlap and Catton, 1994), and that greenness is to protect the environment itself. In the corporate understanding of ‘greenness,’ being green is re-articulated as a way of consuming: consumption is turned to be environmentally responsible and problem-free as long as people consume the green products. In addition, as the advertising discourse implies, consuming green is also rational (because it helps to reduce cost and protect health) and modern (because it has the advanced technologies and is able to solve current environmental threats). Therefore, greenness in green products is not simply a responsibility anymore, but an attraction for consumers. The green products in advertisements are more like a new choice for living a particular lifestyle—a consumption lifestyle. As a means to the ends of living a green lifestyle, to protect the environment is not an end anymore.

This lifestyle is not necessarily related to reducing over-consumption, but a new approach to consumption and a way of extricating both consumers and the consumerism society from environmental worries, although the environmental threats remain. This can be explained as a reflection of social ideological thought of ecological modernization (Hajer, 1995; Coffey and Marston, 2013) and is connected to social change toward post-materialism (Inglehart, 1981).

In addition to the common discursive strategies above, differences can be found among the discursive strategies from the two categories of firms.

### Discursive and Socio-Cognitive Differences

Applying Kress and van Leeuwan’s (2006) visual analysis framework on reading the advertising images from aspects of angle, distance, and size, it can be found that differences existing between advertisings of Chinese indigenous firms (CF) and their counterparts with Western background (WF): WF advertising visuals tend to naturalize the green products while CF ones tend to centralize the green products. Naturalization is achieved by both distance and size visual strategies. With regards to distance, the WF firms either position green products and natural objects in an equal position (see Exhibit 6: Unilever), or position natural objects in a closer position than products, to viewers (see Exhibit 7: GE). Similarly, the natural objects appear to be a larger size than products in WF firms’ green advertisings. Conversely, CF firms apparently give prominence to products instead of natural objects by positioning products in the middle and closer to viewers, and by presenting products in a larger size (see Exhibits 8, 9). Such differences also signify the different relationships between green products and nature/environment: WF advertisings treat products and nature as equal and relate green products closely to the natural environment while CF advertisings value products more highly.

**Exhibit 6:**
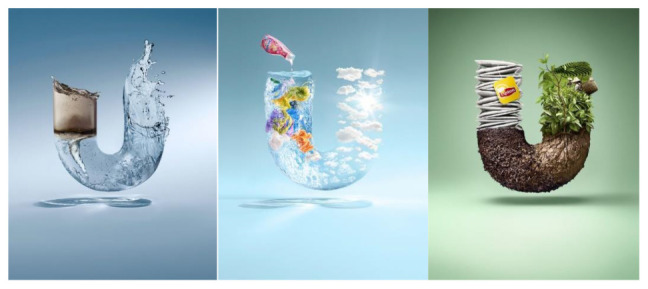
Unilever’s Green Advertisements. Website address: https://www.unilever.com.cn/sustainable-living/. Image is from companies’ public advertisement online and the permission of publication of advertising images has been obtained.

**Exhibit 7:**
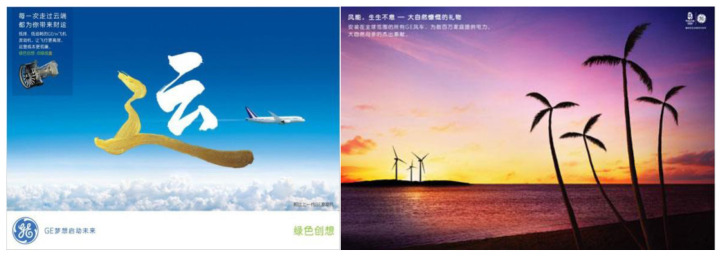
GE’s Green Advertisements. Website address: http://www.ge-energy.com/wind. Image is from companies’ public advertisement online and the permission of publication of advertising images has been obtained.

**Exhibit 8:**
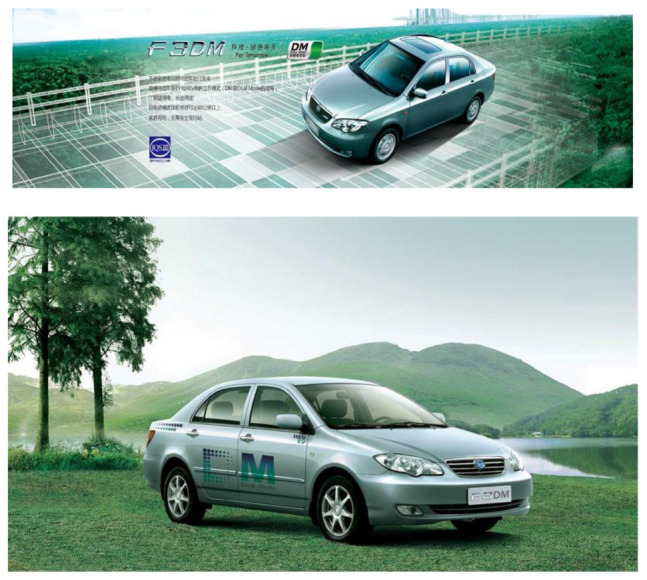
BYD’s Hybrid Cars. Website address: http://www.bydauto.com.cn/car-360-F3DM.html. Image is from companies’ public advertisement online and the permission of publication of advertising images has been obtained.

**Exhibit 9:**
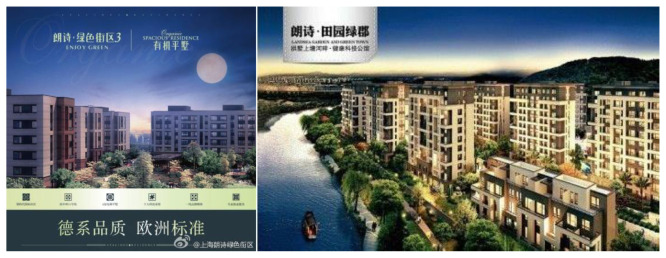
Landsea’s Green Advertisements. Website address: http://landsea.cn/Group/RealEstate.aspx. Image is from companies’ public advertisement online and the permission of publication of advertising images has been obtained.

Chinese indigenous firms online advertising discourse for their green brand appears to highlight the companies’ spirit of altruism and philanthropy as their corporate responsibility practices, which inform an environmentalism discourse. Meanwhile, in the case of green product discourse, it is interesting to find that the previously mentioned corporate concern for environmental protection or the environmentalism discourse has largely disappeared and been replaced by other discourses such as the functional, utilitarian, and economic. Furthermore, the environmental protection value of corporate greenness seems to be placed in a peripheral position and becomes simply decorative in the composition of the product advertisement. In this sense, the discourse seems to be self-contradicting.

Such paradoxical discourse found in the advertisings cannot be simply concluded as ‘greenwash.’ In Lyon and Maxwell (2011)’s definition, ‘greenwash’ refers to ‘the selective disclosure of positive information about a company’s environmental or social performance, without full disclosure of negative information on these dimensions, so as to create an overly positive corporate image’ (p. 5). In the example of this study, however, the corporate green discourse is not found to hide negative information such as side-effects^10^ of the corporate greenness. Instead, what it does is it purposely highlights certain kinds of discourse in certain areas. For example, as mentioned above, utilitarian and functional facets of greenness are placed as important, as the feature of environmental protection. This discursive arrangement and strategy appear different from that found in MNCs (WF)’ green advertisings.

Different from the Chinese firms’ highlighted and advertised philanthropy practices, MNCs (WF)’ green advertising discourse presents a more ‘developmental’ approach with emphasis on the concept of ‘sustainability’: this concept not only stresses eco-efficiency and environment impact reduction, but also places emphasis on growing business and increasing consumption. Moreover, the linguistic novelty such as coinage of new green words and terms (e.g., GE’s ecomagination, Unilever’s Sustainable Living Plan) reflects a proactivity in WF firms which have integrated environmental protection into their business development strategies. For example, GE’s Ecomagination and Unilever’s Sustainability Living Plan both represent a strategic purpose which not only talks about protecting the natural environment but also makes business sense. Such linguistic novelty can be seen to demonstrate an intention for active change. Moreover, WF advertising discourse expresses environmental responsibility as intrinsically motivated. In WF advertisements, it is not mainly the outside pressures or appeals leading to corporate green turn, but also, and more importantly, the companies’ understanding of the business opportunities in future green market.

In comparison to the proactive stance, Chinese firms in their green discourse are passive: CF advertising discourse attributes their environmental responsibility partly to the governmental and civil demand, and thus external political appeals, For example, Landsea states:

*‘In the next 10 years, facing the macro environmental upheavals such as economic slowdown and growing attention for environment from both government and the society, Landsea will rely on its green reputation and technologies and keep walking on the path of ‘deep green*.’^11^

In addition, even though both categories try to portray themselves as ‘green leaders,’ the CF firms present themselves as the first leaders to acclaim and respond to the governmental appeal for becoming green, while the WF firms present themselves as leaders establishing and addressing international environmental standards and world-leading environmental practices. This signals different political orientations: CF discourse seeks to gain green acceptance and legitimacy, while WF discourse establishes their authority and governance by importing industrial standards and practice codes. Compared with CF’s follower role answering the state’s environmental call, WF’s stance is more proactive, to give one example: ‘*We are not satisfied with meeting with the governmental regulations and laws in environmental issues. As market leaders, we have higher standards for our own practices.*’

In short, differences exist in green advertising discourses and they differ on two aspects (see Table 3): the aspect of ***greenness dimensions*** and the aspect of ***greenness solutions***. In the aspect of dimensions, WF is more focussed on environmental protection function than CF in terms of its environment-centered/concerned discursive configuration. In the aspect of solutions, WF is more proactive with integrating environmental responsibility in alliance with business opportunities and development strategies. Besides, under the theme of ‘sustainability,’ WF is enacting sustainability norms and environmental practices in their green advertising discourse. Such sustainability discourse can be seen as a manifesto of MNCs to take political and leadership roles in environmental governance. In comparison, CF is treating environmental protection mainly as a response to governmental demands in a passive stance.

**TABLE 3:** Differences between green advertising discourses from western background (WF) and Chinese indigenous firms (CF).

**Dissimilarities**		**WF: MNCs**	**CF: Indigenous Firms**
**Aspect of**		Object-oriented	Context-oriented
**greenness dimensions**	Green vocabulary	Coinage of new green terms such as ‘ecomagination’ (GE), ‘Sustainability Living Plan’ (Unilever); consistently presenting the environmentalism focus	No newly coined green words; Vocabularies from multiple domains (environmentalism, technocratic, and politics)
	Focus	To manifest the environmental friendliness of the product; Dematerializing the product	To highlight the functionality of the product (e.g., energy reduction and high performance); Marginalizing the environmental factors; instrumental view; More anthropocentrism
**Aspect of greenness solutions**	Features	strategic, organizational Sustainability: reservation/protection and business growth	public, relational Responsibility: emphasizes the responsibility for environment (national pride, patriotism, promote national environment, harmony, and prosperity)
	Political orientation	Environmental governance -Proactive	Environmental legitimacy -Passive

*MNCs, multinationals.*

In addition, a strategy-oriented approach toward green business is found in WF’s green discourse surrounding the idea of ‘sustainability.’ The term of sustainability development is better articulated and integrated by considering environmental protection and meanwhile making business sense: for GE, sustainability means that green is green: green business brings dollars. For Unilever, under the umbrella idea of the Sustainability Living Plan, the advertising discourse interprets sustainability on one hand as consuming environmentally friendly products to a green lifestyle, and on the other hand as opportunity for growing the business. In such discursive approach, the term of sustainability development is made comprehensible and provides guidance for practices.

Compared with the MNCs green discourse, it seems that the Chinese corporate green discourse is more oriented toward a culture of benefits – emphasizing the practicality of greenness for the consumers while erasing the green benefits for the environment. The co-existence of this pragmatist attitude and philanthropy feature as one of the indigenous characteristics of Chinese corporate environmental responsibility is influenced by the emerging market economy in China, and is closely related to China’s social and cultural backgrounds (Xu and Yang, 2010).

### Explaining the Differences

The differences (see Table 3) existing in the studied corporate green discourses mark the contextual influences shaping the construction of greenness and the objective of environmental protection.

In the aspect of greenness dimensions, the MNCs’ green advertising discourse is object oriented and solely focusing on environmental protection, while Chinese ones show a context-oriented approach. Such differences can be further explained by probing into the contextual backgrounds. First, in the realm of socio-cognitive systems, Chinese people would be expected to seek compromise solutions to problems based on principles of holism/dialectics and continuity, and to try to reconcile seeming contradictions (Nisbett et al., 2001). Therefore, the greenness dimensions appear to be more inclusive with a focus on not only environmental protection, but also product functionality and political awareness. In contrast, driven by analytical thinking and an object-oriented approach, firms with a Western background show a proactive stance toward environmental protection. They begin to treat solutions to environmental problems as an opportunity further prospering their business and allying greenness with strategies. While Chinese firms appear to be more passive to ecological improvement with business management in marketplace, instead, they rely on and show consistency with instructions and standards from government. Such discursive difference coincides cognitive difference: Chinese are collectivist and oriented toward the group and authorities, whereas America and other European-influenced societies are more individualist (Nisbett et al., 2001). Moreover, Chinese tend to hold a passive attitude in dealing with environment while Westerners an active and conquering attitude (Chiu, 1972).

In addition, according to Fei (1992), the way that Western societies are organized is like the way people collect straw: the straw is firstly bound into small bundles and then several bundles are bound into larger bundles; and then these bundles are stacked. By making an analogy between organizations in Western societies and the composition of haystacks, Fei (1992) indicated that in Western society the people in an organization form a group, and their relationship to the organization is usually the same. If there are differences among group members or distinctions among ranks within the organization, these would have been agreed upon as part of the rules of the organization. An individual may join several organizations, but it is impossible for a straw to be in several bundles at the same time. That is the difference between people and straws. Fei’s purpose in making the analogy is to see more concretely the pattern of personal relationships in social life, what he henceforth calls the “organizational mode of association” (tuantigeju).

In comparison to the Western way of organizing patterns, Fei (1992) argues that the Chinese pattern is unlike distinct bundles of straws. Instead, it is somewhat like the circles that appear on the water surface of a silent lake when a rock is thrown into it. Each person has a social position at the center of his/her social circles produced by his or her own social influence. Meanwhile, each one’s social circles are interrelated. For Chinese, their most important relationship – kinship – is like the concentric circles. This pattern of organization functions in Chinese traditional society and still largely remains in the modern Chinese mind. In the circle-like networks that make up the Chinese mindset, a self at the center of each web always exists, and the self-centered mindset is not aligned to individualism, but egocentrism.

With individualism, individuals make up organizations in the same way that parts make up the whole. The balance between parts and whole produces a concept of equality: since the position of each individual in an organization is the same, one person cannot encroach on the others. It also produces a concept of constitutionality: an organization cannot deny the rights of an individual; it controls individuals merely on the basis of the partial rights they have willingly handed over. Without these concepts, such organizations as these could not exist. However, in Chinese traditional thought, there is no comparable set of ideas, because there is only egocentrism for Chinese people.

Similarly, Confucian ethics are linked to the idea of discrete centers fanning out into a web-like network. As Confucius wrote, “What the superior man seeks is in himself; what the petty man seeks is in others.” With sentiments such as these, Confucius could not be like Jesus, who so loved everyone under the sun, including his enemies. These actions could not have been motivated by egocentrism. It is often thought that the Chinese would sacrifice their organizations or parties for their own self-interests or families’ interests, their country for their party’s and organizations’ interests, and the whole world for their country’s interests. Accordingly, environmental protection as a public and outer circle interest is thus sacrificed for an individual’s personal inner circle interests.

Now it is clear about the boundary between the public and private spheres: only the “self-interest” is satisfied, he or she can consider its next interests sitting on outside circle relationship; only the interests of people in his or her inner circle relationship met, he or she can begin to think about the next circle. Scarifying the family for individual member’s interests, or the lineage for the interests of one’s household, is in reality a formula. This formula explains the fact that the civic consciousness as well as environmental responsibility is much less developed in China than Western society.

Second, in addition to the socio-cognitive perspective explanation, in the political realm, China did not experience a powerful and consistent environmental movement as did the United States and Europe. Instead, Chinese politics have been deeply influenced by Confucianism, which proposes strict social hierarchy and demarcates the responsibilities of the ruler and the subject. This belief extricates ordinary Chinese citizens from concerns about public issues such as environmental problems (Weller, 2006). Under Mao’s reign, the socialist egalitarianism and Mao’s call on the Chinese to participate in collective actions eventually coalesced into a collective violence against nature (Shapiro, 2001). Post-Mao Chinese society relapsed into the Confucian tradition and citizens again became indifferent toward public affairs. Most citizens believe that environmental protection is the government’s business (Weller, 2006; Zhang and Dran, 2002).

Also in China, the state regime directs and coordinates institutional change, i.e., the Chinese Communist Party and the state administrative bodies are the rule-makers, and others bodies such as companies and non-government organizations follow the rules (Tsai, 2001; Child et al., 2007). The dominant and repressive role that the state plays in constructing regulatory pillars for the system of environmental protection, and the follower’s role played by the Chinese indigenous companies, helps to shape the discursive features of their green advertising. For example, CF firms’ green advertising discourse bears a ‘political accent’ (compared with WF firms’ business strategic orientation) and attaches much importance to government policies and regulations regarding environmental protection and responsibility.

Factors from the economic realm play another role in restraining the constitution of green norms. While the Western consumers have achieved an elevated place in post-industrial life and begun to pursue post-materialism consumption such as green consumption (Ger and Belk, 1996), China’s bourgeoning capitalism has not reached the stage of mass consumerism which has paved the road for green consumption in the West. China’s rapid economic development polarizes the society into the poor and the rich. The poor, comprising most of the population, are still struggling to enhance their very low living standards. As users but not consumers, they seek to fulfill needs from commodities’ functions. Although China’s rising middle class is influenced by the imported environmentalism from the West through media and education, their green consumption for a green lifestyle is comparably in an early stage and their demand for expensive status-symbols such as green products is limited.

## Discussion and Conclusion

Given the findings reviewed above, the research can now summarize the findings. It is found that in representing their greenness, firms apply both textual and visual languages to construct corporate green hegemony and maintain their power by developing a corporate environmental discourse.

To be more specific, in a CDA lens, the descriptive analyses suggest that firms shape themselves as environmentally responsible and authoritative, and represent themselves as taking a green leader’s position. Based on the interpretative analyses, the themes as the ‘meaning tissues’ of green consumption have been identified. Firstly, a recurring theme in corporate greenness discourse is the subversion of subjects: the corporate discourse reframes consumers as non-commercial. A particular subject that companies frame and align with their targeted consumers is the value-advocate/contributor/participant/patron of responsible and savvy practices. The semantic power in advertising discourse frames audiences’ interpretations that they are not just consumers, but also have other subjective positions. In such a way, the promotional consumption discourse produces the possibility of an ‘ideal self’ of consumer culture (Thompson, 2004) and implies to the audience that being a consumer can also be a contributor in environmental protection, as long as consumers choose the advertised green products. Secondly, the object of ‘consuming green’ is structured as being morally superior to the ‘other.’ Such constructed dualisms and built boundaries also play a role to mythologies and idealize the greenness in the green products/firms (Thompson, 2004). Indeed, the discursive practices of corporate green advertising rely on a mixture of these elements to imbue the green consumption with meanings and make it interpretable.

In conclusion, compared with the ‘deep green’ advocated by environmental activists, the advertised greenness is the ‘in-breadth green,’ which helps to balance consumerism with environmental conservation. Therefore, the components in green products are presented both horizontally via interdiscursivity and vertically via intertextuality. Such a process of meaning-making has been enabled through both ‘intertextual’ (e.g., newly produced texts are from fragments of existing, conventional ones) and ‘interdiscursive’ (e.g., texts are drawn from texts from other domains of discourses) properties of discourse, and enables the audience to draw upon a wider range of social-historical backgrounds.

There are three theoretical contributions in this study. Firstly, this research extends the corporate environmental responsibility literature by showing how firms are discursively constructed through their commercial green advertisings. Secondly, linked into consumer culture theory and by accepting that corporations are capable to influence the meaning of environmental responsibility, this study advances a critical understanding of how firms influence the nature, meaning, and knowledge of environmental consumption. Thirdly, this research contributes to literature of green marketing by finding out how green advertising practices vary and identifying the characteristics of green advertisements in a developing country context.

### Divergence and Localization of Commercial Green Discourse

Although the transnational advertising industry tries to spread a universal version of green consumerism around the world, this universalizing scheme is found to be localized by local firms in their green marketing and advertising (Li, 2010). This shows that advertising, in spite of its dazzling visual power and excellent outreach capability, is not fully transferred across borders. Instead, the green advertising discourses are embedded in a society’s particular cultural-historical and institutional conditions and thus cannot be universalized.

As Corbett (2006) suggests, ‘the social construction of nature or the definitions and meanings, which people tend to build through social interaction about nature, can be quite different from culture to culture,’ and furthermore, all environmental messages ‘have ideological roots that are deep and that are influenced by individual experience, geography, history, and culture’ (Corbett, 2006, p. 6). Based on such points, firms are expected to adjust their environmental messages to their target audience, especially via the use of green advertising. And the representation of the ‘greenness’ constructed by firms operating in China (corporate environmental responsibility practices, environmental features of products/services) is likely to be influenced by Chinese contexts.

The analysis findings have also indicated differences exist between MNCs’ and Chinese firms’ green discourses. Such finding supports the argument that the globalization process of environmentalism is not homogeneous or unitary (Weller, 2006). And the globalization of environmentalism as well as green discourse is not simply a diffusion process from a single core to the rest of the world. Instead, the green discourse is influenced by contexts (socio-cognitive systems, historical and political backgrounds) but still bears specific characteristics. As in the light of Fei’s theory, the anthropocentric and pragmatic feature of green discourse found in Chinese corporate green advertising can be understood as a reflection of the pattern of organization in Chinese society and the egocentric culture and mindset.

In conclusion, although firms all have their communication channels to shape their discourse and influence, the communication discourse, such as green advertisings, is subject to external constraints from existing social structures (Chouliaraki and Fairclough, 1999). In addition, this study adds a point: besides external influence, internal resource (such as firm’s experiences and operation networks) also helps a discourse turn into hegemonic intervention.

This research finding suggests that, in order to improve firms’ integrated environmental practice and innovation, it is necessary to develop a comprehensive approach within the organization, as well as a holistic and systematic perspective and supporting ecosystem among stakeholders (Cocca and Ganz, 2014; Yan et al., 2018). As green innovation and sustainability development involve multiple stakeholders (Banerjee, 2003), a collaborative ecosystem (government-academia-industry) needs to be adopted and connected to green consumption: firstly, a nationwide macro viewpoint is necessary for the planning of green innovation norms and environmentally friendly industrial developments, and the idea of collaborative ecosystem should be embedded in governmental and public policies. Secondly, once governmental authorities and agencies take environmental impact and sustainability development model as an important criterion of national/regional economy, the green innovation/technology of industrial practices can be better assessed and encouraged. An appropriate green innovation index system can also be established during this process. Thirdly, an industrial clustering mechanism can be developed and additionally facilitate the green industrial practices to enhance the level of sustainability development. In the end, a feedback structure is essential for identifying the critical success factors and trends of consumer expectations. Based on the identified information, firms conducting green innovation are able to better construct their green advertising content. The commercial green discourse therefore can deepen green consumption – helping to enhance consumers’ environmental awareness as well as the value of green innovation products.

### Implications, Limitations, and Future Research

The research findings have several implications for practices. Firstly, the analysis on corporate discourse has revealed the conflict between environmental protection and resource preservation, and the intrinsic exploitative nature of capitalist business. So, this implication suggests integrating economics into its environmental, social, and political integument. Partial reforms are totally insufficient. What is needed is to replace the single micro-rationality of profitability criterion with an environmental and social macro-rationality, which means that civilization will have to operate according to a different paradigm.

In order to achieve greater sustainability and more substantive progress, a number of elements of marketing thought and practice need to be reshaped for industrial and marketing practitioners. Firstly, the advertiser should encompass the means of production and the broader activities of the producer. This can help consumers base their purchase decision on issues beyond the tangible products. Secondly, the markets need to be changed. New types of market in which material flows become more circular through product recycling, and alternative forms of production and consumption (e.g., farmers’ markets) can be created or rediscovered. Thirdly, marketers and advertisers can emphasize more on the benefits from product use rather than on the joys of product ownership. And marketing and advertising communication can aim to inform rather than just impress. The agenda for change is radical and challenging for marketing practitioners and industry. However, without addressing these issues, marketing will continue to act as an obstacle to progress toward genuine sustainability. In addition, it needs combined efforts of consumers, practitioners, policy makers, and scholars and educators.

This study has several limitations which present opportunities for future research. First, the current study is based on a sample of advertisements from websites of four companies that operate in China. The robustness of the findings could be tested by continuing to collect advertisements over time and with more companies’ green advertisements. This study has explored and identified the differences among the firms which are from different backgrounds, following this direction in the future studies, the data scale can be further enlarged. Moreover, as this study findings suggest, there are correlations between discourse and its contexts, it is important to find out more details on the differences in the advertisings’ cultural, historical and societal contexts, and how the differences exert influence on the localization processes of multinationals’ advertising discourse. Besides focusing on the relationship between corporate discourse and its external context, it is also interesting to consider the organizational internal influences on its green discourse.

Secondly, this study is limited to online advertisements. Future studies could include different advertising mediums applied by companies, such as printing and television advertisements, to compare their discursive constitutive characteristics.

Last, as recent branding research suggests that brand meanings are co-constructed through a dialog between managers and consumers (Roper et al., 2013), future research can also extend the managerialist paradigm to incorporate the consumer, and to identify the dialectic, co-constructed nature of environmental consumerism culture.

## Data Availability Statement

The datasets generated for this study are available on request to the corresponding author.

## Author Contributions

SL developed the analytic framework and worked on literature review, data collection, data analysis, and manuscript writing. XL worked on data analysis and literature review. Both authors contributed to the article and approved the submitted version.

## Conflict of Interest

The authors declare that the research was conducted in the absence of any commercial or financial relationships that could be construed as a potential conflict of interest.

## Footnotes

1 http://www.bydauto.com.cn/energy.html 2017/11/23.

2 http://landsea.cn/Group/RealEstate.aspx; accessed 2017/11/23.

Accessed 2017/11/18.

Accessed 2017/11/18.

Accessed 2017/11/18.

Accessed 2015/10/9.

Accessed 2015/10/10.

8 http://www.unilever.com.cn/sustainable-living/; accessed 2015/11/12.

9 http://landsea.cn/About/Citizens.aspx; accessed 2015/11/8.

10 An example of “side-effect” can be found in some case that firms reported electric utility reductions in their greenhouse gas emissions but their actual emissions rose.

11 http://landsea.cn/About/Value.aspx?id=4; accessed 2015/10/10.
